# Sexual Harassment in the House of Medicine and Correlations to Burnout: A Cross-Sectional Survey

**DOI:** 10.31486/toj.19.0019

**Published:** 2019

**Authors:** Eva Mathews, Rebecca Hammarlund, Rumneet Kullar, Lauren Mulligan, Thanh Le, Sarah Lauve, Carine Nzodom, Kathleen Crapanzano

**Affiliations:** ^1^ Our Lady of the Lake Medical Center Psychiatry Residency Program, Louisiana State University, Baton Rouge, LA; ^2^ Department of Academic Affairs, Our Lady of the Lake Medical Center, Baton Rouge, LA; ^3^ Department of Psychology, Louisiana State University, Baton Rouge, LA; ^4^ New York State Psychiatric Institute, Columbia University Medical Center, New York, NY

**Keywords:** *Burnout–professional*, *female*, *medicine*, *physicians*, *sexual harassment*

## Abstract

**Background:** Burnout is a major problem among physicians in the United States. Women physicians experience higher rates of both burnout and sexual harassment than their male counterparts. Some studies from Asia and Europe have shown a correlation between sexual harassment at work and burnout in women physicians, but no studies on this topic have been done in the United States.

**Methods:** For this study, women physicians with active Louisiana licenses were invited to complete a cross-sectional self-report survey to assess burnout and sexual harassment. Burnout was assessed with the 2-item Maslach Burnout Inventory, and sexual harassment was assessed with a questionnaire adapted from the Sexual Experiences Questionnaire and a series of follow-up items.

**Results:** The survey response rate was 13% (129 of 970 invitees). Of the 129 participants, 36% reported feeling burned out from their work at least once a week and 38% reported having experienced at least one inappropriate sexual incident in their career. Ninety-six percent of respondents reported having experienced gender harassment from their colleagues, while 69% had experienced unwanted sexual attention from the same. Additionally, 69 (53%) participants reported experiencing some form of sexual harassment from patients or their families. Colleague gender harassment was significantly correlated with burnout scores.

**Conclusion:** This study found that reports of burnout and gender harassment from colleagues were significantly correlated. The results also align with previous findings of high rates of sexual harassment in medical school and residency. More research should be done in this area, especially focusing on women in training, women of color, and sexual and gender minority individuals.

## INTRODUCTION

Burnout is a major problem among physicians in the United States. In a 2018 national poll of more than 15,000 physicians, 42% reported burnout.^1^ Burnout is not benign, as increased levels of burnout overlap with and may influence levels of depression and suicide.^2^ In addition to having negative consequences for the burned-out physician, burnout has been related to poor patient outcomes.^3^

Burnout may be defined several different ways. In this study, we used 2 facets of Maslach and Jackson's definition of burnout: emotional exhaustion (feeling emotionally drained, fatigued, and unable to connect to patients on a psychological level) and depersonalization (feeling callous towards people, treating patients as impersonal objects).^4^

Recent research indicates gender differences in physician burnout. A 2016 study of internal medicine residents found that women experienced emotional exhaustion more often than their male peers.^5^ In the Medscape National Physician Burnout & Depression Report 2018, nearly half of female physicians expressed feeling burned out vs 38% of their male peers, and lack of respect from administrators/employers, colleagues, or staff was one of the top 3 contributors to burnout.^1^ Addressing correlates and potential causes of burnout is a crucial endeavor, as burnout has been shown to negatively impact patient care and colleague/staff interactions.^1^

The #MeToo movement opened the conversation about sexual harassment in a variety of professional fields, including medicine. Broadly, sexual harassment consists of sexual assault or coercion, unwanted sexual attention, and gender harrassment.^6^ Many studies have identified the onset of sexual harassment in medical school, which can result in deleterious professional outcomes for victims, including reduced career advancement, reduced confidence in themselves professionally, and even leaving the field during training and beyond.^7-10^ Psychological sequelae associated with sexual harassment include isolation, guilt, anger, disrupted sleep, nightmares, fear, depression, stress disorders, and suicidal thoughts.^9,11,12^ A 2018 report from the National Academies of Sciences, Engineering, and Medicine in the United States noted that sexual harassment has not declined in the last 30 years and identified gender harassment as the most common type of sexual harassment.^6^ Examples of gender harassment include comments about women not belonging in leadership positions, demeaning comments about women, sexual insults, and crude sexual jokes. Gender harassment devalues women in the workplace by communicating the idea that women do not belong or merit respect, and such harassment may be related to the higher burnout rates among women physicians.

A study from Japan found that women faculty at a large academic institution experienced higher rates of direct and indirect sexual harassment than their male counterparts (indirect harassment was defined as witnessing harassment of someone else), and for women, indirect harassment was associated with higher rates of burnout than direct sexual harassment.^13^ A study of physicians in Sweden and Italy showed that women physicians with suicidal thoughts, a common correlate of burnout, during the prior year were more likely to have experienced degrading experiences or harassment at work.^11^ Some studies of physicians in the United States have shown that sexual harassment in the workplace is correlated with reduced career satisfaction, but these studies did not specifically measure burnout.^7,12,14,15^ To our knowledge, no studies in the United States have examined the relationship between sexual harassment and burnout in female physicians.

The primary purpose of this study was to address this gap in the literature and to identify the relationship, if any, between sexual harassment and burnout in female physicians with active licenses who are practicing in Louisiana. We also wanted to examine the relationship between sexual harassment of female physicians by colleagues vs by patients and their families. We hypothesized that sexual harassment would be associated with female physician burnout.

## METHODS

The study instrument was a cross-sectional, online, self-report survey. The study was approved by the Louisiana State University Health Sciences Center New Orleans Institutional Review Board.

### Participants

We purchased a database of male and female physicians with active state of Louisiana medical licenses from the Louisiana State Board of Medical Examiners (n=17,352). The names of physicians who were marked in the database as retired or working in other states were removed (n=4,852). Next, researchers coded each name for sex, as this information was not included. We conducted internet searches to attempt to classify androgynous names. All male names (n=8,729) were removed, leaving 3,771 names that appeared to belong to females. Each name was assigned a random participant number from 1 to 3,771, and an online random number generator was used to create a list of 1,000 numbers within this range. The researchers used Google to recheck the list of 1,000. In this search, 5 potential participants were identified as males. Those names were removed and were replaced by 5 randomly selected additional participant numbers.

### Measures

#### Demographics

The first page of the survey contained 9 items. The first item asked for the participant number from the postcard (refer to the Procedure discussion). The remaining items asked for sex, year of birth, race, ethnicity, marital status, career stage (if currently in residency or year graduated from residency), primary medical specialty (from a dropdown list of 25), and current payment model.

#### Burnout

Burnout was assessed with the 2-item Maslach Burnout Inventory (MBI), a validated measure of burnout that assesses emotional exhaustion and depersonalization.^4,16,17^ For both items, respondents are asked to rate the frequency on a scale ranging from “never” to “every day.” High emotional exhaustion and high depersonalization were defined by a frequency of feeling emotionally drained or feeling callous toward people at least once a week.

#### Sexual Harassment

Sexual harassment was assessed with a questionnaire adapted for the current study and a series of follow-up items about any instances of sexual harassment that respondents had officially reported at their institutions.

### Sexual Experiences Questionnaire

The Sexual Experiences Questionnaire (SEQ)^18^ has been described as the gold standard measurement tool for sexual harassment,^19^ but it is more a series of somewhat related surveys than a single standardized measure.^20^ Various versions of the SEQ have been used that include different numbers of items as well as differently worded items, instructions, and response options.^20^ Nevertheless, the original SEQ contains a number of face-valid items related to inappropriate sexual experiences and gender harassment.

We adapted the version of the SEQ found in Kearney^21^ for this study (Table 1). The Kearney SEQ^21^ begins with 2 items that ask if the respondent has ever been sexually harassed and how the respondent was harassed. We did not include these items in our survey, as other open-ended items were used to probe the same topics. Next, the Kearney SEQ^21^ contains 19 items that measure gender harassment, unwanted sexual attention, and sexual coercion. We preserved these 3 subscales for our study, but we combined or deleted items to reduce redundancy (eg, 2 items about unwelcome touching were combined), reducing the number of items to 13. In addition, we slightly reworded some items so they made more sense with the response options. For example, one item on the Kearney SEQ^21^ stated “frequently made sexist remarks…,” which was redundant with the frequency response options (eg, can one “often” “frequently make sexist remarks”?). For our study, we deleted the word “frequently,” so the item stated, “made sexist remarks….” Table 1 presents the original and the modified text for each survey item.

**Table 1.** Original Text of and Changes Made to the Sexual Experiences Questionnaire Published in Kearney^21^

Item		Deleted/	Modified/		
Number	Original Text	Maintained?	Not Modified	Modified Text	Comments
	Initial 2 items:Have you ever been sexually harassed while at the university? YES or NOHow were you harassed?	Deleted	N/A		These items were excluded in favor of our own open-ended items probing this issue.
1	habitually told suggestive stories or offensive jokes?	Deleted	N/A		This item was deleted because it is redundant with item 3.
2	made unwanted attempts to draw you into discussion of personal or sexual matters (eg, attempted to discuss or comment on your sex life)?	Maintained	Not modified		
3	made crude and offensive sexual remarks, either publicly (eg, in the office) or to you privately?	Maintained	Not modified		
4	treated you “differently” because of your sex (eg, mistreated, slighted, or ignored you)?	Maintained	Modified	put you down, mistreated, slighted, ignored, or was condescending towards you because of your sex?	Combined text from items 4 and 9; all behaviors by which one could be treated differently because of sex were combined into one item. “Treated you ‘differently’” was deleted and “because of your sex” was retained to create one item from two redundant items.
5	gave you unwanted sexual attention?	Maintained	Not modified		
6	displayed, used, or distributed sexist or suggestive materials (eg, pictures, stories, or pornography)?	Maintained	Not modified		
7	frequently made sexist remarks (eg, suggesting that women are too emotional to be scientists or that men should not be the primary caretakers of children because they are not nurturing)?	Maintained	Modified	made sexist remarks (eg, suggesting that women are too emotional to be scientists or that men should not be the primary caretakers of children)?	“Frequently” was deleted because it was redundant with the response options, while “because they are not nurturing” was deleted to shorten the item as the phrase is superfluous.
8	attempted to establish a romantic relationship with you despite your efforts to discourage this person?	Deleted	N/A		This item is redundant with item 10, and item 10 is the more precisely worded of the two items.
9	“put you down” or was condescending to you because of your sex?	Deleted	N/A		Combined with item 4.
10	has continued to ask you for a date, drinks, dinner, etc, even though you have said “no”?	Maintained	Modified	continued to ask you for a date, drinks, dinner, etc, even though you have said “no”?	Deleted the auxiliary verb has.
11	made you feel like you were being subtly bribed with some sort of reward or special treatment to engage in sexual behavior?	Maintained	Modified	made you feel like you were being subtly bribed with some sort of reward (eg, faster promotion) or special treatment to engage in sexual behavior?	Combined text from items 11 and 16; item 16 was used an example within item 11 as the two items were redundant.
12	made you feel subtly threatened with some sort of retaliation for not being sexually cooperative (eg, the mention of an upcoming evaluation, review, etc)?	Maintained	Modified	made you feel subtly threatened with some sort of retaliation for not being sexually cooperative (eg, the mention of an upcoming evaluation or review, or implying you would be treated poorly)?	Combined text from items 12 and 18 and added text by the authors; item 18 was used as an example within item 12, with which it was redundant; “or” was added twice and “implying” was inserted to make the combination make more sense.
13	touched you (eg, laid a hand on your bare arm, put an arm around your shoulders) in a way that made you feel uncomfortable?	Maintained	Modified	made unwanted and uncomfortable attempts to touch, stroke, or fondle you (eg, touching your arm or hand, stroking your leg or neck, etc)?	Combined text from items 13 and 14 as both are about unwelcome/uncomfortable touching and added text by the authors to better combine the items.
14	made unwanted attempts to stroke or fondle you (eg, stroking your legs or neck, etc.)?	Deleted	N/A		Combined with item 13.
15	made unwanted attempts to have sex with you that resulted in you pleading, crying, or physically struggling?	Maintained	Not modified		
16	implied faster promotion or better treatment if you were sexually cooperative?	Deleted	N/A		Combined with item 11.
17	made it necessary for you to respond positively to sexual or social invitations in order to be well-treated on the job or at school?	Maintained	Modified	made it necessary for you to respond positively to sexual or social invitations in order to be well-treated on the job?	The words “or at school” were deleted.
18	made you afraid you would be treated poorly if you didn’t cooperate sexually?	Deleted	N/A		Combined with item 12.
19	treated you badly for refusing to have sex?	Maintained	Not modified		

Notes: Response options for this study were “never,” “1 or 2 times in my career,” “sometimes in my career,” “often in my career,” and “very often in my career.” Response options for the original Kearney Sexual Experiences Questionnaire were “never,” “once,” “sometimes,” “often,” and “very often.” Gender harassment was assessed by items 1-4, 6-7, and 9; unwanted sexual attention by items 5, 8, 10, and 13-14; and sexual coercion by items 11-12 and 15-19.

In addition to shortening the scale, we adapted the response options so they were relevant to the respondent's entire medical career. The original SEQ^21^ used the response options “never,” “once,” “sometimes,” “often,” and “very often.” Our response options were “never,” “1 or 2 times in my career,” “sometimes in my career,” “often in my career,” and “very often in my career.”

We adapted the survey instructions to differentiate between 2 different sources of the harassment behaviors. The SEQ – Colleagues version asked about individuals at work as behavior sources, specifying that “individuals” referred to “faculty, physician colleague, nurse, pharmacist, etc.” The SEQ – Patients and Their Families version specified the source of the behavior as a patient or patient's family member.

Finally, in addition to the adaptations described above, we added item 14 asking if the respondent had been mistaken for a nonphysician care provider. We considered this item a face-valid signal of subtle gender bias (eg, the assumption that a female in a healthcare setting is not a physician).

The survey instruments used in the study are shown in the Figure.

**Figure.**
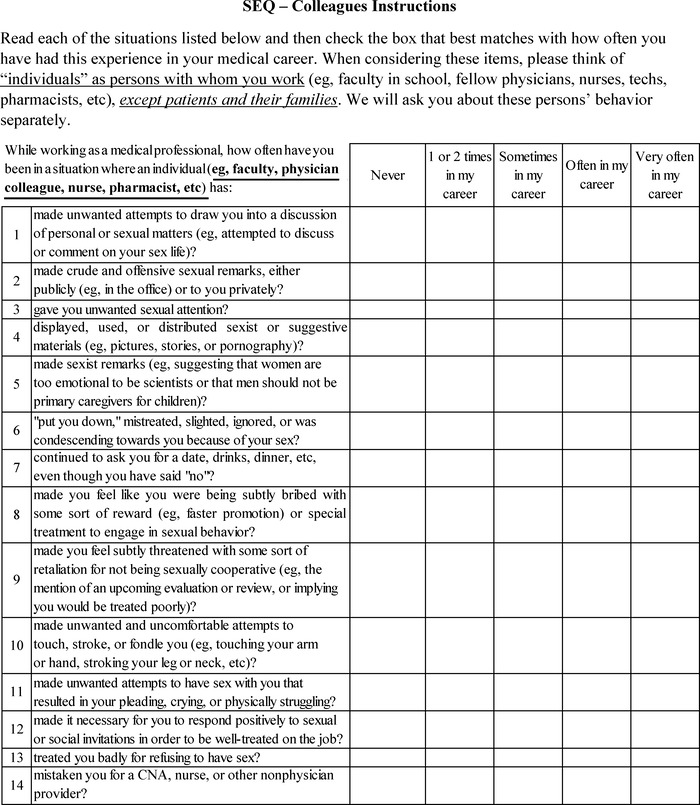

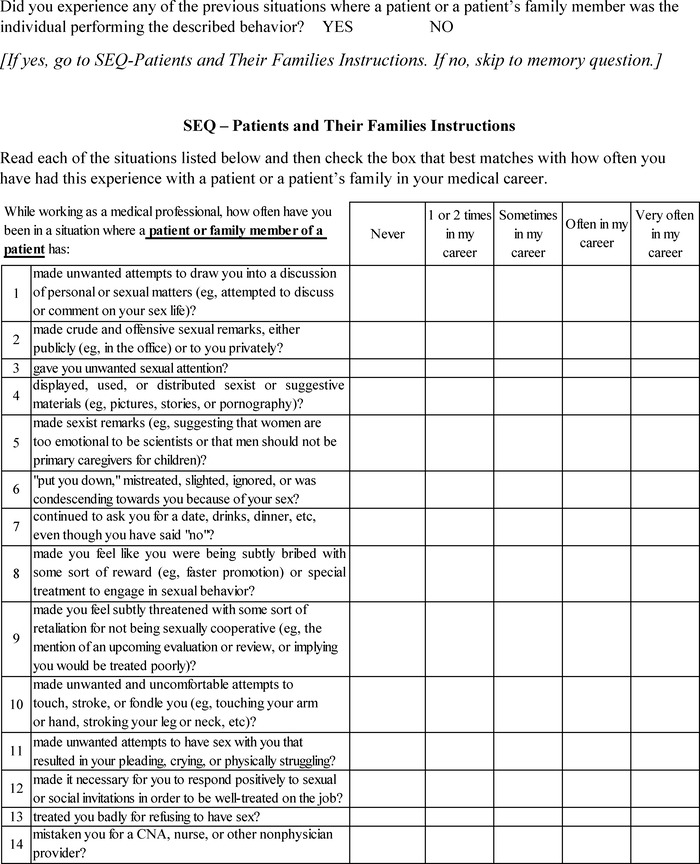
**Adapted Sexual Experiences Questionnaire (SEQ) used to ascertain sexual harassment experiences and frequencies from both colleagues and from patients and their families.** CNA, certified nurse assistant.

### Follow-Up Items

The first follow-up item asked if the respondent had experienced an inappropriate sexual incident that stood out in her memory. Those who answered yes were asked (1) how many such incidents they had reported (if none, the remaining follow-up items were skipped), (2) if they felt the report(s) had been handled appropriately, (3) when the most serious incident occurred, (4) perceived severity of the incident, and (5) if they would like to share their story.

### Procedure

A postcard invitation to participate was sent to the physical address of each of the 1,000 randomly selected physicians on July 16, 2018. The invitation contained a brief description of the study, the link for the online survey, and the invitee's random participant number. Upon following the link, invitees viewed an information page that provided the usual components of an informed consent document and stated that clicking to continue indicated consent to participate. Respondents completed all measures in the order described above. All items had the option to not answer.

Two weeks after the original invitation was sent, researchers used the participant numbers that respondents had entered in the survey to remove those who had already participated from the invitation list. Reminder postcards were sent to the remaining invitees. In addition, we used internet searches to obtain email addresses for as many invitees as possible because only physical addresses were included in the original database. We sent email invitations to those for whom we found an email address, which was principally individuals at large institutions such as universities and academic medical centers. During the process of preparing reminders, we removed the names of 30 invitees from the list because they were discovered to be out of state (n=25), male (n=3), retired (n=1), or a member of the research team (n=1). Reminders were not sent to these individuals, so 970 of the original 1,000 invitees were eligible to participate.

The survey remained open until August 31, 2018, at which time the data were downloaded. Once the data had been cleaned and participant numbers were checked against the invitation list, the link between participant names and participant numbers was destroyed to ensure confidentiality.

## RESULTS

### Respondents

Of 970 eligible invitees, we received 136 (14%) responses. However, 5 respondents were removed from data analyses because they reported being retired, 1 was removed because she did not answer any of the sexual harassment items, and duplicate responses were removed for a participant who responded twice. The final participation rate was 13% (129 of 970 invitees). All respondents were female, and 10 (8%) were residents. Mean time since residency graduation was 12.72 ± 9.62 years (range, 0 to 38 years). Mean age (current year minus reported birth year) was 44.40 ± 10.86 years (range, 28 to 75 years). Table 2 presents the other demographic details for the sample.

**Table 2.** Demographics of Survey Respondents (n=129)

Variable	n (%)
Race	
White/European American	95 (74)
Black/African American	14 (11)
East Asian/Asian American	8 (6)
South Asian/Indian American	6 (5)
Native American/Alaska Native	1 (1)
Mixed race	5 (4)
Ethnicity	
Not Hispanic/Latinx	125 (97)
Hispanic/Latinx	4 (3)
Marital status	
Single	21 (16)
Married	96 (74)
Divorced	6 (5)
Widowed	6 (5)
Payment model	
Employed	108 (84)
Self-employed	19 (15)
No response	2 (2)
Specialty category	
Medical	86 (67)
Surgical	8 (6)
Mixed medical-surgical	27 (21)
No patient care	6 (5)
No response	2 (2)

### Inappropriate Sexual Incidents

In all, 49 (38%) respondents reported having experienced an inappropriate sexual incident that stood out in their memory. Of these, 33 (67%, or 26% of the total sample) did not officially report the incident. One woman wrote that she did not report because the perpetrator was the person to whom she would have to report the incident. Of the 16 women who reported at least one incident, only 3 (19%) felt their reports were always handled appropriately. In contrast, 8 (50%) felt that none of their reports were handled appropriately, and 5 (31%) felt that only some of their reports had been handled appropriately.

### Burnout

Forty-six (36%) respondents reported feeling burned out from their work at least once a week (2-item MBI item 1, representing emotional exhaustion), while 7 (5%) said they never felt burnout. Thirty (23%) respondents reported being more callous towards people at least once a week (2-item MBI item 2, representing depersonalization), while 22 (17%) reported never experiencing callousness. Twenty-three (18%) respondents reported experiencing both symptoms at least once a week, and 6 (5%) reported never experiencing either symptom.

### Sexual Harassment

Table 3 presents descriptive statistics for the 3 subscales of the adapted SEQ and item 14 (eg, mistaken role) for both the SEQ – Colleagues and the SEQ – Patients and Their Families versions of the survey. Sixty-nine (53%) respondents answered both surveys. Although we adapted the SEQ for this study, the Cronbach alpha for the subscales was comparable to that reported elsewhere.^18^ Table 3 also presents the percentage of respondents who reported the minimum score for each subscale and item 14 of the adapted SEQ.

**Table 3.** Descriptive Statistics for the Adapted Sexual Experience Questionnaire (SEQ)

						Percentage With
Respondents	Adapted SEQ/Subscale	Minimum	Maximum	Mean ± SD	α	Score of Zero
All respondents (n=129)	**SEQ – Colleagues**					
	Gender harassment	0	20	6.78 ± 4.22	0.84	4
	Unwanted sexual attention	0	12	2.00 ± 2.18	0.82	31
	Sexual coercion	0	14	0.51 ± 1.71	0.86	83
	Mistaken role	0	4	3.15 ± 0.96	N/A	1
Subset that responded to both SEQ – Colleagues and SEQ – Patients and Their Families (n=69)						
	**SEQ – Colleagues**					
	Gender harassment	0	20	8.07 ± 4.18	N/A	1
	Unwanted sexual attention	0	12	2.38 ± 2.24	N/A	17
	Sexual coercion	0	14	0.71 ± 2.16	N/A	80
	Mistaken role	1	4	3.38 ± 0.82	N/A	0
	**SEQ – Patients and Their Families**					
	Gender harassment	0	20	8.06 ± 3.96	0.85	3
	Unwanted sexual attention	0	8	2.90 ± 2.16	0.71	10
	Sexual coercion	0	11	0.46 ± 1.76	0.88	87
	Mistaken role	1	4	3.55 ± 0.72	N/A	0

N/A, not applicable.

Gender harassment was assessed by survey (Figure) items 1-4, 6-7, and 9; unwanted sexual attention by items 5, 8, 10, and 13-14; and sexual coercion by items 11-12 and 15-19.

In terms of colleague sources of harassment behavior, respondents who had never experienced gender harassment, unwanted sexual attention, and being mistaken for a nonphysician were in the minority, whereas the majority of respondents had not experienced sexual coercion. Overall, 99% of respondents had been mistaken for a nonphysician by colleagues. In the subset of individuals who had experienced harassment from both colleagues and from patients and their families, 100% had been mistaken for a nonphysician by both colleagues and by patients and their families.

### Colleagues vs Patients and Their Families

Among respondents who completed both surveys, the mean score for colleague unwanted sexual attention (mean 2.38 ± 2.24) was significantly lower than patient/family unwanted sexual attention (mean 2.90 ± 2.16), *t*(68)=**–**2.06, *P*<0.05. Colleague sexual coercion (mean 0.71 ± 2.16) was significantly higher than patient sexual coercion (mean 0.46 ± 1.76), *t*(68)=2.03, *P*<0.05, but patients and their families (mean 3.55 ± 0.72) more often mistook participants for nonphysicians than colleagues did (mean 3.38 ± 0.82), *t*(68)=**–**2.11, *P*<0.05.

### Associations Between Burnout and Gender Bias

Table 4 shows the correlations among the adapted SEQ subscales, item 14, and burnout item sum scores. Only colleague gender harassment and colleague mistaken role (item 14) were significantly correlated with burnout scores.

**Table 4.** Correlations Between Burnout and Adapted Sexual Experiences Questionnaire Subscales

	Sum of	
	Burnout Items	
Adapted SEQ/Subscale	*r*	*p*
**SEQ – Colleagues**		
Gender harassment^a^	**0.19**	0.03
Sexism items	**0.21**	0.02
Sexual items	0.14	0.12
Unwanted sexual attention	0.12	0.18
Sexual coercion	-0.01	0.89
Mistaken role	**0.20**	0.02
**SEQ – Patients and Their Families**		
Gender harassment^a^	0.20	0.11
Sexism items	0.20	0.11
Sexual items	0.17	0.17
Unwanted sexual attention	0.06	0.61
Sexual coercion	-0.15	0.21
Mistaken role	-0.01	0.94

^a^Items in the gender harassment subscale were divided into 2 categories for analysis. Sexist remarks and poor treatment due to gender were summed into sexism items, and sexual discussion, remarks, and materials were summed into sexual items.

*p,* probability value; *r,* correlation coefficient.

We divided items in the gender harassment subscale into 2 categories for analysis. Sexist remarks and poor treatment because of gender were summed into “sexism items,” while sexual discussion, remarks, and materials were summed into “sexual items.” Only colleague “sexism items” was significantly correlated with burnout.

### Standout Incidents

Of the 49 respondents who reported a standout sexually inappropriate incident, 38 provided the year the incident happened. Subtracting the year of the incident from the year of the survey, 20 of the 38 incidents (53%) happened in the past 15 years, 16 (42%) in the past 10 years, and 12 (32%) in the past 5 years. The mean time since the incident was 14.21 ± 11.01 years. The year of standout incident was also compared to year of residency graduation. Four incidents were reported by individuals currently in residency. Of the remaining 34 incidents, 8 (24%) occurred after residency graduation. The remaining 26 (76%) incidents occurred in the same year as residency graduation or earlier.

## DISCUSSION

The purpose of this study was to determine if female physician burnout is correlated to sexual harassment experiences from either colleagues or from patients and their families. Prior research has demonstrated that subjects of sexual harassment are at greater risk for symptoms such as anxiety, sadness, irritability, anger, sleeplessness, and weight loss, as well as major depression and posttraumatic stress disorder.^22,23^ Sexual harassment could, therefore, also impact the symptoms of burnout. We found that reports of burnout and gender harassment from colleagues were significantly correlated, largely attributable to items probing the frequency of sexist remarks and poor treatment because of gender from colleagues. The frequency of colleagues mistaking respondents for nonphysicians was also significantly correlated with burnout. In contrast, gender harassment from patients and their families was unrelated to burnout, as were unwanted sexual attention and sexual coercion from either colleagues or from patients and their families.

One interpretation for this differential pattern of results is that disrespectful behaviors of patients and their families are easier for female physicians to cope with psychologically than disrespect from colleagues. Even though both gender harassment and being mistaken for a nonphysician were reported as occurring approximately equally as often from colleagues and from patients and their families (Table 3), only colleague gender harassment and mistaken role were significantly correlated with burnout. The lack of relationship between burnout and unwanted sexual attention or sexual coercion is likely related to the relative frequencies of the 3 types of sexual harassment. Consistent with previous literature, gender harassment was more frequently reported than the other 2 types of harassment in our study.^18,24^

In the subset that responded to both SEQ – Colleagues and SEQ – Patients and Their Families (n=69), 68% (n=47) of respondents experienced unwanted sexual attention from colleagues, whereas 90% (n=62) reported unwanted sexual attention from patients and/or their families. These findings support the idea that colleagues and patients and their families view female physicians in a less than professional light—as both unlikely to be a physician because of their gender and as an object of sexual interest. Considering these findings, it is perhaps less than surprising that a small but startling minority of respondents overall had experienced sexual coercion from colleagues (17%), and in the subset of respondents who answered both surveys, 13% reported experiencing sexual coercion from patients and their families. While both unwanted sexual attention and sexual coercion were unrelated to burnout in this study, this lack of relationship should not be overinterpreted. Particularly in the case of sexual coercion, the current study was likely underpowered to detect any effects, given the low percentage reporting it. Additionally, burnout may not be the most appropriate construct to measure the impact of such experiences on female physicians. Thus, these reports should not be dismissed. Rather, they suggest that medicine is not isolated from other industries that have recently come under fire for their approach to dealing with claims of sexual harassment and sexual assault.

The moderate to large correlations between the adapted SEQ subscales from colleague and patients and their families sources are consistent with prior literature^18^ and suggest that some individuals have such experiences more frequently than others. This finding could be interpreted as a reporting bias, such that the respondents in this study were more likely than other women to subjectively judge experiences as inappropriate or offensive.^25^ Our study also has the potential for a sampling bias, such that women who have been harassed may have been more likely to participate. However, Berdahl provides evidence to suggest that women in medicine may indeed experience more sexual harassment than other women.^25^ First, Berdahl showed that some women, specifically those with more “masculine personality traits” such as independence, ambition, and analytical ability,^6^ experienced more sexual harassment, and this finding was not attributable to a bias on their part to judge potentially inappropriate situations more negatively than other women.^25^ Additionally, Berdahl showed that women with “masculine traits” are at even higher risk of experiencing sexual harassment in workplaces that are male dominated.^25^ Because medicine continues to be male dominated, it is perhaps of little surprise that sexual harassment experiences seem to be common in medicine.

Our results also align with previous findings of high rates of sexual harassment in medical school and residency in that our respondents reported incidents occurring during their years of training. Surveys used for the National Academies of Sciences, Engineering, and Medicine sexual harassment report showed that 45% to 50% of medical students experienced sexual harassment, whereas the rate was only 20% to 42% for students in other postgraduate fields.^6^ Some prior work suggests why the rate of sexual harassment is so high in medicine. For example, Baillien et al identified several factors operating at the level of the job, the team, and the organization that increase the risk of sexual harassment and other types of bullying or violence in the workplace.^26^ Among these factors are contact with third parties, gender ratios consisting of more men than women, and too strict (or too lenient) hierarchies, all of which apply in medicine.

In medicine, patients and their families are third parties. Employees who are in contact, particularly actual physical contact, with third parties are at higher risk for sexual harassment.^26^ Thus, medical students, residents, and physicians at all levels are more at risk of sexual harassment experiences than individuals in many other professions.

The gender ratio in a workplace is a top risk factor for sexual harassment.^26^ Specifically, sexual harassment is more frequent in workplaces with a greater number of men than women. Despite the increasing number of women entering medicine, leadership positions and certain specialties remain predominantly male. This uneven representation may result in the persistence of inappropriate gender-based expectations and gender power disparities. In other words, differences in gender socialization, such that males are rewarded for assertive behavior and females are encouraged to avoid confrontation, may be one mechanism that perpetuates dominance and submissiveness in the workplace.^22^ Additionally, because of sociocultural factors, males may feel more entitled to positions of power (especially within male-dominated workplaces), while females are expected to fill assistant/helper roles.^22^ These factors may result in the male dominance that has been observed in leadership positions and certain fields of medicine. The results include limiting female physicians’ occupational and economic growth, as well as their vertical mobility via hindrance of entry into leadership positions.

In addition, experienced physicians are employees of high power, both because they may occupy leadership positions and because of their monetary value to their organizations. The total cost of recruiting a new physician has been estimated to be $400,000 to $1,200,000 per physician.^27-29^ This cost may motivate organizations to protect physicians from facing the consequences of misconduct, and in turn, the physicians themselves may come to believe that workplace rules do not apply to them.^30^ Such a mindset means that employees of high value may be at greater risk of becoming perpetrators of sexual harassment or other inappropriate behavior. In contrast, medical students, residents, and early-career physicians have relatively little power in their organizations. They may not challenge conduct that makes them uncomfortable, including unwelcome sexual overtures.^26^ Furthermore, victims of sexual harassment may be concerned that their statements will not be taken seriously or that they will face retaliation, particularly if the perpetrator is a high-value employee the organization will likely wish to protect. Not surprisingly, the Equal Employment Opportunity Commission found that 75% of workplace harassment is never reported.^30^

Several study limitations are worth mention. The principal limitation of our study is the low response rate; we attribute that in part to the lack of email addresses available and, thus, having to use postcards to provide the link to the online survey. The generalization of these results to all female physicians may be limited by our decision to restrict our survey to female physicians in the state of Louisiana. Sampling bias may also limit the results as those who responded to the survey may have had an increased interest in the subject matter and/or have been harassed, thus inflating the prevalence rate. Future research can attempt to obtain a higher response rate and less risk of sampling bias by inviting only participants with known email addresses and/or considering the use of social media for responses.

Second, the magnitude of associations between our adapted SEQ subscales and burnout largely fell within the small effect size range. This finding may stem from the aforementioned low response rate. However, significant associations between our adapted SEQ subscales and burnout are likely not merely spurious or trivial. Burnout is a complex, multifaceted construct that is influenced by a number of variables, including sexual harassment. To our knowledge, our study findings are among the first to suggest that sexual harassment plays an influential role in burnout in medicine. Future research can expand on our findings by exploring potential mediating and moderating variables between sexual harassment and burnout.

Research on sexual harassment of women of color and of sexual and gender minority individuals (eg, gay, lesbian, transgender) is incomplete. Current research shows that these minority groups experience more sexual harassment than white or heterosexual women.^6^ The majority of our respondents were white non-Latinx physicians, and we did not collect information on sexual identity, so we could not evaluate the findings in these minority populations.

We found that those who reported sexism and sexual harassment from patients were at increased likelihood of reporting parallel experiences from colleagues with significant correlations among all of the subscales. This finding appears to support the notion that women who report one form of sexism or harassment have likely experienced other forms. Investigation into how medical leadership is responding to these situations should be explored.

## CONCLUSION

Sexual harassment, which includes gender harassment, is present in medicine and occurs more frequently during the training period. Gender harassment from colleagues, as well as colleagues failing to recognize female physicians as such, contributes to physician burnout. Medical education leadership and administrators need to consider how to protect young learners and address the culture within medicine that allows these behaviors to occur.

## Notes

This article meets the Accreditation Council for Graduate Medical Education and the American Board of Medical Specialties Maintenance of Certification competencies for Medical Knowledge and Professionalism.
